# Dietary Exposure and Health Risk Assessment of Selected Toxic and Essential Metals in Various Flavored Dairy Products

**DOI:** 10.1007/s12011-025-04676-0

**Published:** 2025-06-04

**Authors:** Aml S. Ibrahim, Shimaa S. Awaad, Heba A. Shehta, Ola W. Hegab

**Affiliations:** 1 Department of Food Hygiene and Control, Faculty of Veterinary Medicine, Cairo University, Giza, 12211 Egypt; 2 Regional Center for Food and Feed, Agriculture Research Center, Giza, Egypt

**Keywords:** CR, EDI, Flavored dairy products, Non-carcinogenic risk, Trace elements

## Abstract

Children are strongly frequent consumers of flavored dairy pro ducts chosen and encouraged by their mothers to achieve nutritional requirements. Therefore, this study was implemented to assess flavored dairy products (UHT milk, pasteurized milk, milk powder, yogurt, drinking yogurt, and ice cream) with two diversified flavors for each product for their trace element levels (Al, Pb, Hg, Cd, As, Sb, Zn, Fe, Cu, B, Se, Ni, Co, Cr, and Mn) using inductively coupled plasma-mass spectrometer (ICP-MS/MS). Zn, Cu, Fe, Mn, Co, and Al were detectable in all examined samples, but they were found to be free of mercury and cadmium. Essential elements (Zn, Cu, Fe, Co, Cr, Mn, B) and Al were maximally determined in chocolate milk powder with mean values of 20.73 ± 0.703, 6.832 ± 0.114, 17.9 ± 4.75, 0.299 ± 0.026, 1.279 ± 0.52, 7.958 ± 0.201, 3.51 ± 0.431, and 4.785 ± 1.590 mg/kg, respectively, while Ni, Pb, and As were utmost measured in strawberry yogurt at 1.591 ± 1.746, 0.09 ± 0.036, and 0.037 ± 0.031 mg/kg, respectively. The Pb value concentrations in samples of strawberry milk powder and pasteurized milk exceeded the regulative limit permitted by the European Commission (0.02 mg/kg). Regarding estimated daily intake (EDI) of toxic metals, strawberry drinking yogurt showed Pb (0.0046 mg/kg_bw_/day) and Al (0.164 mg/kg_bw_/day) twice daily intakes for toddlers surpassed the tolerable daily intake (TDI). Therefore, the consumption of strawberry yogurt for toddlers showed a possibility of non-carcinogenic risk expressed as THQ and carcinogenic risk (CR) in minimum to maximum values of 1.870–3.740 and 8.40 × 10^−4^–1.68 × 10^−3^ respectively. The contamination of flavored dairy products should be of greater concern as it is deemed a health crisis. Restrictive control measures must be applied starting from hygienic measures related to dairy animals, harvesting, and preparation of unprocessed materials used in the manufacturing of dairy plants, as the steps of processing and raw material quality have the greatest attention and influence on metal concentrations variability.

**Supplementary Information:**

The online version contains supplementary material available at 10.1007/s12011-025-04676-0.

## Introduction

Flavored milk and dairy products are of high consumption and concern among infants and children worldwide [1, 2]. They are characterized by high percentages of macro- and micronutrients as their plain counterparts, but the addition of flavoring agents and sugar has made them more palatable and attractive. These products provide children with nutritional elements, especially vitamins and minerals, offering bodies health neediness and benefits for improving them mentally as well as physically [3, 4]. Consequently, mothers, for their children’s nourishment, take refuge depending on flavored dairy products, including UHT milk, pasteurized milk, milk powder, yogurt, drinking yogurt, and ice cream. Therefore, manufacturers were encouraged to manufacture these products in a variety of flavors, including chocolate, vanilla, strawberry, banana, and peach, with different consistencies and textures in accordance with consumer priority [5–7].

Although these dairy products are considered a prime dietary source of macro and essential microminerals, especially during childhood, they may also probably contain unacceptable concentrations of toxic elements [8]. Toxic elements such as Cd, As, Pb, Hg, Sb, and Al possess no biological role, although they have serious toxicity impacts on health through destroying and dysfunction of main body organs [9, 10]. Consequently, they may also link to metabolic disorders, osteoporosis with bone damage, hepatic and renal failure, brain damage with mental illness, developmental system weakness (nervous, digestive, immune, cardiovascular, and reproductive), and multiple others [8, 11, 12]. These toxic elements are widely distributed in the earth’s crust and pertinent to industrial processing; therefore, they have evolved as persistent contaminants in the environment and food chain [13].

Despite Fe, Zn, Cu, Cr, Co, and Se being essential trace elements, as well as Mn, Ni, and B being probably essential trace elements, they all perform an indispensable function for individual wholesomeness; their exaggerated intake may give rise to unfavorably detrimental effects on healthiness [14–16]. Therefore, consumption of foods with elevated concentrations of Ni may be related to depression, mental confusion, and hypertension, while Cu and Mn may be associated with psychological flaws such as Alzheimer’s [14, 17, 18]. Several studies reported that various metals have exceeded the definite limit in milk and dairy products [19–22].

Assessing milk products quality by analyzing trace metals contents is highly useful [23]. One of the critical concerns among researchers is the determination of elements dietary intake and comparing it with tolerable intakes set by regulatory authorities as well as the calculation of their carcinogenic slope factor and THQ (target hazard quotient) for evaluation of probable harmful risks to consumer health [24, 25]. The metal concentration in flavored dairy products and their daily intake, especially for a consumer group aged ≤ 5 years, were infrequently conducted in prior studies for ice cream [26, 27], drinking yogurt [23, 28], flavored milk [19, 29], and fruit yogurt [5, 23, 30], without studies for flavored milk powder.

Therefore, the evaluation of the exposure concerning toddlers and children to risk and toxicity from metals through consuming flavored dairy products should be periodically monitored. The current study is designed and proceeded to determine essential, probably essential, and toxic elements in selected dairy products with popular flavors. Daily intake is estimated for both consumer groups, non-carcinogenic and carcinogenic potential risk assessment. Also, comparing is established for these values and metal content with available permissible and regulatory limits.

## Materials and Methods

### Sample Collection and Identification

A total of 180 samples of different dairy products, divided into fifteen samples from each flavor of chocolate (C) and strawberry (S) of pasteurized (PM), UHT, and milk powder (MP); peach (P) and strawberry flavors of yogurt (Y) and drinking yogurt (DY) with chocolate (C) and vanilla (V) ice cream (IC), were randomly collected from Giza governorates, Egypt. All samples were labeled and kept in their packages till analysis at − 18 °C.

### Chemicals and Reagents

Deionized water (Milli-Q water system, 18.2 MΩ cm), nitric acid (69% HNO_3_, Merck, Germany), hydrochloric acid (37% HCl, Merck, Germany), calibration blank of acid solution mixture of (0.5% HCl and 2% HNO3), and the analytical standards of a certified reference material (1000 ppm, Merck, Germany) for each metal were used in the study.

### Sample Digestion and Preparation

The microwave digestion of samples (0.5–1.0 g or ml of each sample depending on moisture content) was done in dry and precleaned appropriate digestion vessels. Then 6 ml of 69% nitric acid (HNO3, Merck-Germany), and 1 ml of Hydrochloric acid 37% (HCl, Merck-Germany) were added, as the samples were digested following the manual instructions in microwave digestion. In addition, gold (0.2 mg/L; Au) was used to help with Hg stabilization. The digestion under high pressure was performed to reach a minimum temperature of 200 °C with a hold of over 20 min and finally maintained for 15 min to achieve accurate digestion. Samples were cooled, filtered, and transferred into per-acid-cleaned vessels, rinsed, and diluted into appropriate volumes (10 ml) using deionized water (Milli-Q water system, 18.2 MΩ cm) based on the working standard with preparation of three blank samples [23, 30, 31].

### Analysis of Metals in Digested Samples

Trace elements (Zn, Cu, Fe, Co, Cr, Mn, Se, Ni, and B) and toxic metals (Pb, Al, As, Sb, Cd, and Hg) were tested using ICP-MS/MS (inductively coupled plasma-mass spectrometer, Agilent 8800 Triple Quad.) as described by primer instructions Agilent 5991-2802EN [32]. The analysis was done in ISO-accredited mineral lab (IEC 17025) in the Regional Center for Food and Feed (RCFF, Agriculture Research Center), Giza, Egypt.

### Quality Assurance and Method Validation

To confirm the reliability of analytical procedures, all reagents utilized were of analytical grade, with suitable and carefully cleaned glassware using nitric acid and deionized water. The instrument was calibrated and tuned to get linear five-point calibration curves that were established using a multi-element standard solution (1000 ppm, Merck). The linearity was confirmed with correlation coefficients (min. 0.99 *R*^2^) for all analytes, and the concentration of the diluted samples was within the calibration range. Internal standards and tuning solutions of the instrument were all supplied by Agilent as recommended by the primer. The quality control sample (QCS 500 ppb, multi-element standard) was purchased and prepared in an acid matrix to ensure matrix compatibility with the dairy sample digestion matrix, and it was used to judge the validation of results and was of acceptable RSD (1 to 5%). A stability graph was achieved using an Agilent internal standard solution with an accepted recovery (90 to 110%). The detection limit (LOD) values were obtained using Agilent MassHunter software (version 4.3) based on the signal-to-noise ratio, while the limit of quantification (LOQ) was measured as 3.3 times the LOD. Each sample was analyzed in triplicate to assess repeatability and precision.

### Exposure Risk Assessment

#### Assessment of Daily Intake

The estimating of the daily dietary consumption of flavored dairy products by children from 1 to 5 years living in Giza, Egypt, was done following Eq. (1), by categorizing into two groups based on children’s age (1–2 and 3–5). While frequency of consumption was classified as follows: once daily and twice a day. Concerning each product, the amount of consumption was calculated based on the purchase amount of each product that was consumed/day as 30 g, 150 g, 200 ml, 220 ml, and 250 ml for milk powder, ice cream and yogurt, UHT-milk, drinking yogurt, and pasteurized milk, respectively**.**

1 $$\mathrm{EDI} = \frac{{\mathrm{FC}}\times {\mathrm{MC}}}{\mathrm{BW}}$$

where estimated daily intake (EDI) of metals (mg/kg_bw_/day), FC, is the amount of food consumption in g or ml/day, MC is metal concentration in each examined product, and BW is the average body weight of two Egyptian population categories: toddlers (10 kg) and preschool children (15 kg) based on WHO [33] and El Shafie et al. [34].

#### Exposure Health Risk (Non-cancer) Assessment

2 $$\mathrm{THQ} = \frac{\mathrm{EDI}}{{\mathrm{RfD}}}$$

where the THQ of metal dietary intake was established by the USEPA [35] using Eq. (2), and RfD (mg/kg_bw_/day) is the reference oral dose of each metal. The RfD of studied trace metals is 0.0035, 0.001, 0.0003, 0.0003, 1.0, 1.5, 0.0004, 0.005, 0.02, 0.3, 0.04, 0.02, 0.7, 0.14, and 0.2 mg/kg_bw_/day for Pb, Cd, As, Hg, Al, Cr, Sb, Se, Ni, Zn, Cu, Co, Fe, Mn, and B, respectively, according to Boudebbouz et al. [25], USEPA [35, 36], Scivicco et al. [37], and El Youssfi et al. [38]. TTHQ of each examined product was determined by summing all THQ metal values for assessment of whole risk following Eq. (3) for every product consumption. The values of THQ or TTHQ more than one may indicate potential health risk; however, values equal to or less than one propose negligible harmful risk on health [25, 37, 38].

3 $$TTHQ\;=\;THQ_{Pb}\;+\;THQ_{As}\;+\;THQ_{Cd}+\;THQ_{Hg}+\;THQ_{Al}+\;THQ_{Sb}+\;THQ_{Cr}+\;THQ_{Se}+\;THQ_{Ni}+\;THQ_{Zn}+\;THQ_{Cu}+\;THQ_{Co}+\;THQ_{Fe}+\;THQ_{Mn}+\;THQ_{B}$$

#### Estimating Carcinogenic Risk

4 $$\text{CR }=\text{ EDI }\times \text{ CSF}$$

where CSF is the oral cancer slope factor calculated based on Eq. (4), which is 0.0085, 1.5, and 1.7 (mg/kg_bw_/day)^−1^ for lead, arsenic, and nickel, respectively [35–37]. The lifetime cancer risk limit is set from 10^−6^ to 10^−4^ as acceptable, although for total carcinogenic risk (TCR) in the same product for multiple elements, it is defined as less than 10^−4^ based on USEPA [35], Scivicco et al. [37], El Youssfi et al. [38], and RAIS [39] using the following Eq. (5):

5 $$TCR\;=\;CR_{Pb}\;+\;CR_{As}\;+\;CR_{Ni}$$

### Statistical Analysis

The data were exhibited in mean ± SD (standard deviation), and the results were statistically analyzed via one-way ANOVA in SPSS 23 [40]. The risk assessment of trace elements exposure from examined flavored dairy products was assessed and calculated based on toddlers’ and children’s daily consumption (one daily serving and twice daily serving).

## Results and Discussion

The metals in examined samples were categorized into three groups: essential trace elements (Zn, Cu, Fe, Co, Cr, and Se), probably essential trace elements (Mn, Ni, and B), and toxic elements (Pb, As, Cd, Hg, Al, and Sb).

###  Trace Elements Incidence in Flavored Dairy Products

**Fig. 1:**
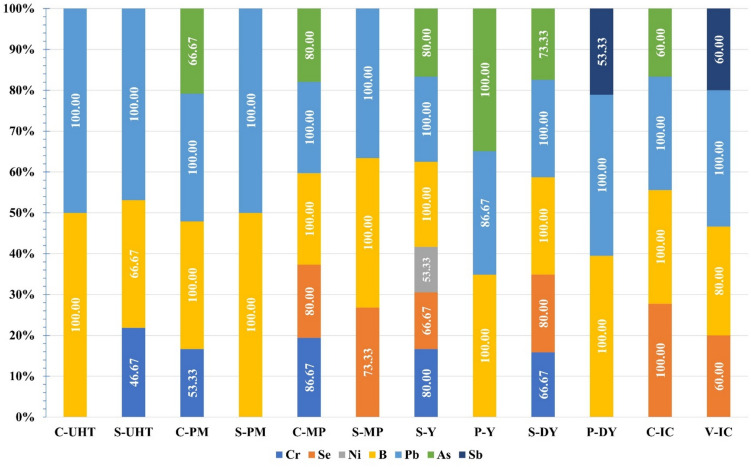
Prevalence of trace metals in flavored dairy products (*n* = 180)

The essential elements represented in Fe, Cu, Zn, Co, and Mn were quantifiable in all studied samples. However, B could be determined in the total samples except in 5/15 samples of S-UHT milk. The Ni was measured at 53.33% of S-yogurt only, but selenium was detected in a percentage of 100.00, 80.00, 80.00, 73.33, 66.67, and 60.00 at C-ice cream, S-drinking yogurt, C-milk powder, S-milk powder, S-yogurt, and V-ice cream, respectively. The highest prevalence of chromium was revealed at 86.67% of C-milk powder, followed by 80% of S-yogurt and 66.67% of S-drinking yogurt (Fig. 1).

The examined toxic element incidence for aluminum was 100% in the examined products, as well as lead, which was determined in all products except peach yogurt in a percentage of 86.67. Cd and Hg concentrations were less than LOD and LOQ values (0.032 and 0.106 ppb for Cd; 0.072 and 0.237 ppb for Hg) in all examined samples, which were also similarly declared by Hameed and El-Zamkan [19], Luis et al. [5], Capcarova et al. [41], Conficoni et al. [27], and Totan and Filazi [29] in various flavored milk products.

Arsenic was measurable in 60.00, 66.67, 73.33, 80.00, 80.00, and 100.00% of C-ice cream, C-pasteurized milk, S-drinking yogurt, C-milk powder, S-yogurt, and P-yogurt, respectively. About 60% of V-ice cream and 53.33% of P-drinking yogurt was contaminated with antimony (Fig. 1).

### Trace Metal Concentrations in Flavored Dairy Products

Both essential and probably essential trace element groups are critical and significant for natural human developmental growth and metabolism, as well as acting as enzymatic cocatalysts [42]. Essential element concentrations averaged in examined products were arranged as follows: Zn, Fe, Cu, Cr, Co, and Se within a range of 3.348–20.73, 1.078–17.9, 0.135–6.823, 0.046–1.279, 0.003–0.299, and 0.010–0.241 mg/kg, respectively. The highest concentrations for all essential metals were determined in chocolate milk powder samples with significant differences (*P* < 0.05) with other products except for selenium in strawberry drinking yogurt. Also, the lowest concentrations were determined in vanilla ice cream but not for iron, which was lowermost determined in strawberry-UHT milk and chromium in chocolate pasteurized milk.

There were non-significant (*P* > 0.05) average values of UHT and pasteurized milk products for Zn, Fe, Co, and Cr; however, they were significantly different from other products in iron concentrations, as presented in Table 1. The zinc permissible limit is 5 mg/kg based on WHO [43], so chocolate-UHT milk, C-pasteurized milk, C-milk powder, C-ice cream, strawberry milk powder, S-yogurt, S-drinking yogurt, and peach yogurt exceeded the limit in average concentrations of 5.491 ± 0.884, 5.88 ± 0.745, 20.73 ± 0.703, 6.172 ± 2.244, 18.14 ± 0.737, 7.011 ± 2.422, 7.937 ± 3.874, and 5.203 ± 0.995 mg/kg, respectively. Consequently, these zinc content data for both fruit yogurt and drinking yogurt are nearly alike to the results obtained by Khan et al. [23] for milk powder and by Oloyede et al. [44] as exhibited in Table 2. Zinc may transfer into milk and its products through machines or tools used throughout production, processing, transportation, and storage [21]. Zn plays an essential and pivotal function in sexual maturation, physiological processes of the immune system, and body performance through its participation as a cofactor in the synthesis and efficiency of almost 300 enzymes [45, 46]. However, higher levels than the allowable limit and chronic zinc exposure may cause impaired absorption of copper and passive impacts on the body, such as immune system malfunctions, leucopenia, and anemia with gastrointestinal disorders [46, 47].

**Table 1:** Concentration (mean ± SD; mg/L or mg/kg) of trace elements in flavored dairy products (*n* = 180)

Products	Essential trace elements						Probably essential trace elements			Toxic elements			
	Zn	Cu	Fe	Co	Cr	Se	Mn	Ni	B	Pb	As	Al	Sb
C-UHT	5.491^a,c,g^ ± 0.884	0.799^a^ ± 0.146	1.278^a^ ± 0.764	0.028^a,e,f^ ± 0.004	ND	ND	1.177^a,e,f^ ± 0.177	ND	1.025^a^ ± 0.110	0.027^a^ ± 0.004	ND	1.345^a,g^ ± 0.468	ND
S-UHT	3.968^b,c,g^ ± 0.641	0.197^b^ ± 0.065	1.078^a^ ± 0.719	0.011^a,e^ ± 0.005	0.117^a,c^ ± 0.130	ND	3.298^b,f^ ± 1.281	ND	0.264^b^ ± 0.259	0.073^b,e^ ± 0.058	ND	0.847^a^ ± 0.341	ND
C-PM	5.88^a,f,g^ ± 0.745	1.121^c^ ± 0.451	2.166^a^ ± 0.954	0.036^a,f^ ± 0.006	0.046^a^ ± 0.045	ND	1.551^a,b,f^ ± 0.317	ND	3.188^c^ ± 0.543	0.028^c,a^ ± 0.004	0.0016^a^ ± 0.001	1.013^a^ ± 0.487	ND
S-PM	4.604^c,g^ ± 0.196	0.356^d,b^ ± 0.07	1.180^a^ ± 0.711	0.013^a,e^ ± 0.007	ND	ND	1.388^a,b,f^ ± 0.067	ND	3.291^c,d^ ± 0.154	0.004^d^ ± 0.003	ND	0.833^a^ ± 0.409	ND
C-MP	20.73^d^ ± 0.703	6.832^e^ ± 0.114	17.9^b^ ± 4.75	0.299^b^ ± 0.026	1.279^b^ ± 0.52	0.022^a^ ± 0.012	7.958^c^ ± 0.201	ND	3.51^d^ ± 0.431	0.075^b,e^ ± 0.014	0.009^a^ ± 0.005	4.785^b^ ± 1.590	ND
S-MP	18.14^e^ ± 0.737	0.451^d,f^ ± 0.199	6.83^c,d^ ± 1.272	0.08^c^ ± 0.051	ND	0.023^a^ ± 0.015	0.724^a,e,g^ ± 0.276	ND	1.602^e^ ± 0.467	0.005^d,a^ ± 0.001	ND	2.591^c^ ± 0.568	ND
S-Y	7.011^f,h^ ± 2.422	1.21^c^ ± 0.752	5.905^c^ ± 2.491	0.199^d^ ± 0.111	0.219^a,c^ ± 0.165	0.012^a^ ± 0.011	3.267^d^ ± 1.808	1.591 ± 1.746	1.151^a,f^ ± 0.290	0.09^e^ ± 0.036	0.037^b^ ± 0.031	2.926^c^ ± 0.771	ND
P-Y	5.203^ g^ ± 0.995	1.122^c^ ± 0.039	6.798^c,d^ ± 2.360	0.032^a,e,f^ ± 0.004	ND	ND	2.6^d,b,f^ ± 0.686	ND	1.625^e^ ± 0.046	0.059^b^ ± 0.026	0.02^c^ ± 0.003	1.985^d,g^ ± 0.859	ND
S-DY	7.937^ h^ ± 3.874	0.612^f,a^ ± 0.126	5.185^c^ ± 2.739	0.018^a,e^ ± 0.004	0.257^c^ ± 0.200	0.241^b^ ± 0.22	2.208^b,f^ ± 1.410	ND	0.45^b^ ± 0.287	0.104^f,e^ ± 0.052	0.0034^a^ ± 0.0025	3.736^e^ ± 1.323	ND
P-DY	3.463^b,c^ ± 1.022	0.222^b^ ± 0.047	6.48^c,d^ ± 3.562	0.007^e^ ± 0.001	ND	ND	0.382^e,g^ ± 0.059	ND	1.418^f,e^ ± 0.596	0.025^c,a,d^ ± 0.0123	ND	4.028^f^ ± 0.979	0.021^a^ ± 0.025
C-IC	6.172^a,f^ ± 2.244	1.475^ g^ ± 0.584	8.430^d^ ± 4.656	0.051^f^ ± 0.017	ND	0.038^a^ ± 0.029	1.767^f,b^ ± 0.786	ND	1.246^a,f^ ± 0.301	0.051^b^ ± 0.042	0.005^a^ ± 0.004	1.79^ g^ ± 0.777	ND
V-IC	3.348^b^ ± 0.955	0.135^b^ ± 0.017	6.064^c^ ± 3.634	0.003^a^ ± 0.0002	ND	0.010^a^ ± 0.009	0.251^ g^ ± 0.044	ND	0.99^a^ ± 0.535	0.068^b^ ± 0.039	ND	0.914^a^ ± 0.307	0.0017^b^ ± 0.0019

*ND* not detected, different letters in the same column indicate significant differences (*P*- value 0.05)

**Table 2:** Trace elements content in dairy products of previously published research articles (mg/kg)

Products	Essential trace elements						Probably essential trace elements			Toxic elements				Study
	Zn	Cu	Fe	Co	Cr	Se	Mn	Ni	B	Pb	As	Al	Sb	
C-UHT	-	-	-	-	-	-	-	-	-	0.108 ± 0.034	0.0038 ± 0.0028	0.984 ± 0.106	-	**Hameed and El-Zamkan **[19]
S-UHT	-	-	-	-	-	-	-	-	-	0.107 ± 0.046	0.0057 ± 0.0070	0.964 ± 0.136	-	
UHT	-	0.62 ± 0.08	-	-	-	-	-	-	-	1.94 ± 0.26	-	-	-	**Mansour et al. **[81]
C-PM	0.362 ± 0.105	0.015 ± 0.011	0.202 ± 0.359	-	0.011 ± 0.006	0.003 ± 0.003	-	ND	-	ND	0.115 ± 0.046	0.004 ± 0.002	-	**Totan and Filazi **[29]
S-PM	0.561 ± 0.231	0.002 ± 0.004	0.083 ± 0.138	-	0.011 ± 0.006	0.002 ± 0.003	-	ND	-	ND	0.114 ± 0.091	0.005 ± 0.003	-	
C-PM	-	-	-	-	-	-	-	-	-	-	-	1.770 ± 0.190	-	**de Paiva et al. **[65]
S-PM	-	-	-	-	-	-	-	-	-	-	-	1.050 ± 0.065	-	
PM	71.25	0.92	4.05	-	-	-	0.58	-	-	0.003	-	-	-	**Zwierzchowski and Ametaj **[82]
PM	4.54	0.06	0.23	-	-	-	0.02	-	-	0.0015	-	-	-	**Astolfi et al. **[83]
PM	3.5–24.0	0.180 ± 0.04	0.50 ± 0.2	-	-	-	ND	-	-	ND	-	-	-	**Oloyede et al. **[44]
MP	-	-	20.41 ± 0.621	-	-	0.014 ± 0.001	0.497 ± 0.030	-	-	0.791 ± 0.057	-	1.57 ± 0.094	-	**Salah et al. **[84]
MP	-	-	-	-	2.70 ± 0.34	-	-	-	-	0.05 ± 0.01	-	-	-	**Abdelkhalek et al. **[63]
MP	-	-	-	-	-	-	-	-	-	0.004 ± 0.0005	0.008 ± 0.001	-	-	**Singh et al. **[85]
MP	6.2–38.2	0.3–0.6	1.5–6.6				0.3–1.5	-	-	0.14–0.21	-	-	-	**Oloyede et al. **[44]
SY	-	-	-	-	-	-	-	-	0.038 ± 0.001	-	-	-	0.006 ± 0.0002	**Altunay and Gürkan **[86]
Flavored-Y	2.47 ± 0.210	0.25 ± 0.07	0.34 ± 0.19	0.002 ± 0.001	0.02 ± 0.01		0.02 ± 0.01	0.01 ± 0.004	0.05 ± 0.02	0.001 ± 0.001	-	0.45 ± 0.270		**Luis et al. **[5]
SY	0.91 ± 0.13	0.21 ± 0.03	-	-	0.36 ± 0.08	-	3.71 ± 0.06	0.19 ± 0.05	-	0.26 ± 0.04	-	-	-	**Capcarova et al. **[41]
SY	-	ND	0.010 ± 0.000	-	ND	-	ND	-	-	ND	-	-	-	**Souza et al. **[87]
Fruit-Y	-	0.059 ± 0.003	-	-	ND	-	-	-	-	0.114 ± 0.0076	-	-	-	**de Andrade et al. **[30]
Fruit-Y	4.334 ± 0.004	0.229 ± 0.0002	-	0.004 ± 0.00001	0.217 ± 0.0002	0.814 ± 0.005	0.298 ± 0.0004	0.133 ± 0.0002	-	0.025 ± 0.00003	0.001 ± 0.00004	-	-	**Khan et al. **[23]
Fruit-DY	3.668 ± 0.008	0.718 ± 0.002	-	0.005 ± 0.00001	0.305 ± 0.0005	0.679 ± 0.005	0.237 ± 0.0004	0.167 ± 0.0003	-	0.013 ± 0.00006	0.0007 ± 0.00004	-	-	
S-DY	-	0.17 ± 0.01	3.17 ± 0.06	-	ND	-	0.09 ± 0.01	0.4 ± 0.04	-	ND	-	-	-	**Abdel-Rahman et al. **[28]
P-DY	-	0.22 ± 0.03	5.27 ± 0.02	-	ND	-	0.12 ± 0.01	1.37 ± 0.12	-	ND	-	-	-	
C-IC	-	2.04 ± 1.830	11.48 ± 8.140	-	-	0.06 ± 0.046	1.49 ± 0.590	0.004 ± 0.010	-	0.61 ± 0.460	0.008 ± 0.002	10.50 ± 9.000	-	**Darwish and Darwish **[26]
V-IC	-	1.490 ± 0.970	9.320 ± 7.280	-	-	0.05 ± 0.041	1.52 ± 1.190	0.074 ± 0.250	-	0.62 ± 0.400	0.001 ± 0.004	15.80 ± 5.190	-	
IC	-	-	-	-	-	-	-	-	-	0.008–0.011	0.013–0.044	4.870–4.920	0.001–0.003	**Millour et al. **[73]
IC	4.23–4.87	1.62–1.74	-	0.036	0.338–0.391	0.04	1.87–1.89	0.341–0.365	-	-	-	-	-	**Noël et al. **[88]
IC	5.87 ± 1.89	1.82 ± 0.56	0.78 ± 0.38	-	0.44 ± 0.10	-	0.46 ± 0.27	0.99 ± 0.23	-	0.36 ± 0.14	-	9.60 ± 2.17	-	**Dhar et al. **[89]

*ND* not detected (below LOD)

FAO/WHO [48] set a maximum limit (ML) of 0.05 mg/kg for copper in milk fat products only and 2 mg/L for drinking water by WHO [49]. Currently, there is no specific limit of Cu included in all dairy products specifically regulated by Codex. The IDF [50] in 1979 had specified an ML for Cu in milk and its products of 0.01 mg/kg. Higher concentrations of copper content were observed in chocolate dairy products, which may have contributed to the copper content of the cocoa powder used. The European Commission [51] announced a maximum limit in 2006 (Cu 0.6 mg/kg) for a final product containing cocoa powder. The chocolate-flavored examined products showed a significantly higher Cu value compared to another product flavor. The legal limit of copper in these products should be clearer and more accurate. The identical consequences were attained by Totan and Filazi [29], who reported that cocoa milk was relatively high in Cu content and frequency. The increased doses of copper were reported to possibly induce gastrointestinal disorders, especially in young children, or may be combined with Wilson’s disease [20, 52, 53]. Although the surpassing intake of the toxic threshold of iron (200 mg) may give rise to organ impairment such as the heart and liver [54].

The uppermost concentrations of iron and cobalt were determined in C-milk powder expressed in mean values of 17.9 ± 4.745 and 0.299 ± 0.026 mg/kg, respectively. The maximum limit specified for Fe and Co was 0.37 mg/kg, referenced to IDF [50], and 0.05 mg/kg suggested by EFSA [55], respectively. The milk powder and S-yoghurt examined samples surpassed the limit of Co with values significantly differing (*P* > 0.05) than other products. However, none of the assayed products exceeded the limit of chromium of 0.3 mg/kg and specified 2 mg/kg for milk powder, as well as not transcending the selenium-defined maximum limit (0.5 mg/kg) [56, 57]. The results attained by Darwish and Darwish [26] were higher in both kinds of tested ice cream than our data for Fe and Cu, with nearly similar concentrations for Se in C-ice cream and high value in V-ice cream. Generally, the prevalence of copper in milk products may be related to Cu-plated equipment, as well as elevated Fe content, which may be related to its liberation from metallic utensils used during manufacturing operations or incorporated from raw material utilized in production [28, 58, 59].

On observance of probably essential trace elements, manganese exhibited the uppermost mean values, ranging from 0.251 ± 0.044 mg/kg of V-ice cream to 7.958 ± 0.201 mg/kg of C-milk powder, plus it displayed a significant difference to other products, followed by boron in minimum to maximum concentrations of 0.45 ± 0.287 mg/kg for S-drinking yogurt to 3.51 ± 0.431 mg/kg for C-milk powder. However, nickel was only determined in a mean of 1.591 ± 1.746 mg/kg in S-yogurt, almost similarly to Abdel-Rahman et al. [28]. Results were of high values of yogurt and drinking yogurt containing fruit parts with Mn, which are nearly similar to Capcarova et al. [41], who reported a mean value of 3.71 ± 0.06 mg/kg in S-yogurt, as well as compatible with the results obtained by Khan et al. [23], who concluded significantly higher Mn and Ni levels in fruit-mixed yogurt and drinking yogurt compared to plain samples.

There was no legislation set by the EU or WHO for Mn, B, and Ni specified for milk products. On the other hand, in 2011, the EU announced a regulated migration limit for Ni and Mn in food of 0.02 and 0.60 mg/kg, respectively [60]. As well as the guideline values established in 2017 by WHO of Mn, B, and Ni for drinking water were 0.4, 0.5, and 0.07 mg/L [49].

Regarding detectable toxic elements, the LOD and LOQ values were 0.0026 and 0.0085 ppb for Pb, 0.015 and 0.049 ppb for As, 0.096 and 0.317 ppb for Al, and 0.023 and 0.078 ppb for Sb. Aluminum was the most contaminated metal detected in examined products, with the lowest concentration in S-pasteurized milk (0.833 ± 0.409 mg/kg) and the highest concentration in C-milk powder (4.785 ± 1.590 mg/kg), followed by lead (Pb) in an average concentration of 0.004 to 0.104 mg/kg. The mean values of assessed products exceeded the recommended maximum limit of 0.02 mg/kg announced by the European Commission [61] except in S-pasteurized milk and S-milk powder. However, elevated values were noticed by Darwish & Darwish [26]; Hameed and El-Zamkan [19]; Capcarova et al. [41].

C-milk powder, Peach, and S-drinking yogurt showed a significant difference in results of Al with other products, as well as the results of S-drinking yogurt samples for Pb, which were significant for tested products except S-yogurt. However, there was no significant aluminum concentration in the chocolate and strawberry milk samples surveyed, which was similar to data shown by Totan & Filazi [29]. The trace elements in the environment may be generated by volcanic explosions as a naturalistic origin and from human activities like water pipelines, pesticides, fertilizers, and more; however, the main source in milk products may be attributed to water and animal feed or packing materials [62, 63].

Referenced to EU and WHO regulations, there is no maximum limit for Al in food. However, the Council of Europe [64] announced a maximum SRL (specific release limit) of 5 mg/kg for the release of Al into foodstuffs. As represented in Table 1, all tested products had mean values less than 5 mg/kg; however, C-milk powder and both drinking yogurt flavors were significantly higher (*P* < 0.05) compared to other products. However, products with chocolate flavor have an aluminum content relatively higher than other flavors, compatible closely with results revealed by de Paiva et al. [65]. The studies conducted by Stahl et al. [66] and Bertoldi et al. [67] reported contamination of cocoa powder with high values of Al 80–312 and 10.6–275 mg/kg, respectively, owing to the nature of Al in the growing soils, which explains the elevated concentration of chocolate dairy products. Khalifa and Ahmad [68] and de Paiva et al. [65] reported contamination of fruit purees, which may be used in fruit fermented milk products preparation, with maximum Al content of 6.450 and 3.450 mg/kg, respectively, as well as a mean value of 3.1 ± 0.1 mg/kg in fresh strawberries by Pereira et al. [69]. Generally, this may be related to pesticide application through cacao and fruit cultivation [29, 70].

Concerning arsenic, the P-yoghurt exhibited a statistically significant difference compared to others. However, none of the contaminated products was reported to have a value higher than the limit (0.1 mg/kg) set in 2011 by Joint FAO/WHO [71] for arsenic. The lowest prevalence of toxic metals was determined for Sb (antimony) in P-drinking yogurt and V-ice cream in mean values of 0.021 ± 0.025 and 0.0017 ± 0.0019 mg/kg, respectively, without surpassing the restrictions (0.04 mg/kg food) applied by EFSA [72]. The previously explained data for antimony in ice cream was nearly conformable to Millour et al. [73]; however, our findings were less for As, Pb, and Al.

Lower contents were observed for As and Pb in flavored milk with higher aluminum concentration than in data recorded by Hameed and El-Zamkan [19]. In addition to that, minor concentrations were obtained for Pb as 0.010 ± 0.002 and 0.008 ± 0.0018 mg/kg in pasteurized milk and yogurt, respectively, by Shahbazi et al. [74]. Recent studies have attributed the contamination of flavored-fruit dairy products via toxic elements to the usage of fruits that may be polluted through contaminated irrigated water and farming soil, in addition to pesticides and fertilizers usage extremely [28, 75, 76]. Also, the diversity of these products in toxic element values may be referred to fruit kind specifically, feed contamination levels, season and locality of milk production, final product processing, packaging, and through conveyance [28, 77, 78]. Generally, several studies attribute various contamination metal levels and kinds in the examined products to procedures technologically applied during their manufacture, such as industrial processing, packing, shipping, and transportation. Consequently, milk and other components become in friction with diversified metallic equipment [5, 79, 80].

### Estimated Daily Intake (EDI) of Elements Through Flavored Dairy Products Consumed by Children and Toddlers

The EDI was calculated for all metals in each product on consumption by two groups (toddlers and children) in two categories of consumption as follows: one serving daily (minimum), which is the daily amount depending on the purchase amount of each pack, and maximum, which is twice the packs. The most elevated daily intakes were determined for zinc (0.349 mg/kg_bw_/day), followed by iron (0.285 mg/kg_bw_/day), nearly identical to Yu et al. [21] and Luis et al. [5], who stated that zinc has the highest EDI value in dairy products generally and specifically for fermented milk products.

Among essential trace elements, the Co showed EDI above the set TDI, as presented in Table 3, for C-milk powder (0.0018 mg/kg_bw_/day), C-pasteurized milk (0.0018 mg/kg_bw_/day), and S-yogurt (0.002–0.006 mg/kg_bw_/day), while S-drinking yogurt for Se for toddlers and children in maximum consuming of 0.011 and 0.007 mg/kg/day, respectively. Owing to Ni in S-yogurt, it surpassed the TDI proposed in 2020 by EFSA [93] for both groups of consumers at two levels of consumption in a range of 0.016–0.048 mg/kg_bw_/day. Toddlers were in danger of consuming S-drinking yogurt twice/day via lead (Pb) toxicity in the range of (0.0015–0.0046 mg/kg_bw_/day), which was identically like data recorded by Sujka et al. [22], as well as by Yu et al. [21] of the highest Pb and Co daily intake in fermented milks. The results of Pb and Cu EDI of fruit yogurt for children recorded by de Andrade et al. [30] were nearly conformable to calculated intake, as displayed in Tables 3 and 4.

**Table 3:** EDI (mg/kg_bw_/day) of essential trace elements through consumption of flavored dairy products (*n* = 180) in comparison to TDI

Products	Consumer	Essential trace elements											
		Zn		Cu		Fe		Co		Cr		Se	
		1/D	2/D	1/D	2/D	1/D	2/D	1/D	2/D	1/D	2/D	1/D	2/D
C-UHT	T	0.11 ± 0.02	0.22 ± 0.035	0.016 ± 0.003	0.032 ± 0.006	0.026 ± 0.015	0.051 ± 0.03	0.0006 ± 0.0001	0.0011 ± 0.0001	-		-	
	C	0.073 ± 0.01	0.146 ± 0.024	0.011 ± 0.002	0.021 ± 0.004	0.017 ± 0.01	0.034 ± 0.02	0.0004 ± 0.00005	0.0007 ± 0.0001	-		-	
S-UHT	T	0.079 ± 0.01	0.159 ± 0.026	0.004 ± 0.001	0.008 ± 0.003	0.022 ± 0.014	0.043 ± 0.029	0.0002 ± 0.0001	0.0005 ± 0.0002	0.0023 ± 0.0025	0.005 ± 0.005	-	
	C	0.053 ± 0.009	0.106 ± 0.017	0.003 ± 0.0009	0.005 ± 0.002	0.014 ± 0.009	0.029 ± 0.019	0.0002 ± 0.0001	0.0003 ± 0.0001	0.0016 ± 0.0017	0.0031 ± 0.0035	-	
C-PM	T	0.147 ± 0.019	0.294 ± 0.037	0.028 ± 0.011	0.056 ± 0.023	0.054 ± 0.024	0.108 ± 0.048	0.0009 ± 0.0002	**0.0018** ± 0.0003	0.0011 ± 0.001	0.0023 ± 0.0022	-	
	C	0.098 ± 0.012	0.196 ± 0.025	0.019 ± 0.008	0.037 ± 0.015	0.036 ± 0.016	0.072 ± 0.032	0.0006 ± 0.0001	0.0012 ± 0.0002	0.001 ± 0.0007	0.002 ± 0.0015	-	
S-PM	T	0.115 ± 0.005	0.23 ± 0.01	0.009 ± 0.0018	0.018 ± 0.004	0.024 ± 0.018	0.049 ± 0.036	0.0003 ± 0.0002	0.0007 ± 0.0004	-		-	
	C	0.077 ± 0.003	0.153 ± 0.007	0.006 ± 0.001	0.012 ± 0.002	0.016 ± 0.012	0.039 ± 0.024	0.0002 ± 0.0001	0.0004 ± 0.0002	-		-	
C-MP	T	0.062 ± 0.002	0.124 ± 0.004	0.020 ± 0.0003	0.041 ± 0.0007	0.054 ± 0.014	0.107 ± 0.028	0.0009 ± 0.0001	**0.0018** ± 0.0002	0.004 ± 0.0016	0.008 ± 0.003	0.00007 ± 0.00004	0.00013 ± 0.0001
	C	0.041 ± 0.001	0.083 ± 0.003	0.014 ± 0.0002	0.027 ± 0.0005	0.036 ± 0.009	0.072 ± 0.019	0.0006 ± 0.0001	0.0012 ± 0.0001	0.003 ± 0.001	0.005 ± 0.002	0.00004 ± 0.00002	0.00009 ± 0.00005
S-MP	T	0.054 ± 0.002	0.109 ± 0.004	0.0014 ± 0.0006	0.0027 ± 0.001	0.02 ± 0.0038	0.041 ± 0.008	0.0002 ± 0.00015	0.0005 ± 0.0003	-		0.00007 ± 0.00004	0.00014 ± 0.0001
	C	0.036 ± 0.001	0.073 ± 0.003	0.0009 ± 0.0004	0.0018 ± 0.0008	0.014 ± 0.0025	0.027 ± 0.005	0.0002 ± 0.0001	0.0003 ± 0.0002	-		0.00005 ± 0.00003	0.00009 ± 0.00006
S-Y	T	0.105 ± 0.036	0.21 ± 0.072	0.018 ± 0.011	0.036 ± 0.022	0.089 ± 0.037	0.177 ± 0.074	**0.003** ± 0.002	**0.006** ± 0.003	0.003 ± 0.002	0.007 ± 0.005	0.0002 ± 0.00017	0.00037 ± 0.0003
	C	0.07 ± 0.024	0.14 ± 0.048	0.012 ± 0.008	0.024 ± 0.015	0.059 ± 0.025	0.118 ± 0.05	**0.002** ± 0.001	**0.004** ± 0.002	0.002 ± 0.0016	0.0044 ± 0.003	0.00012 ± 0.0001	0.00025 ± 0.0002
P-Y	T	0.072 ± 0.015	0.144 ± 0.03	0.017 ± 0.0006	0.034 ± 0.001	0.102 ± 0.035	0.204 ± 0.071	0.0005 ± 0.0001	0.0009 ± 0.0001	-		-	
	C	0.048 ± 0.01	0.096 ± 0.02	0.011 ± 0.0004	0.022 ± 0.0008	0.068 ± 0.024	0.136 ± 0.047	0.0003 ± 0.00004	0.0006 ± 0.0001	-		-	
S-DY	T	0.175 ± 0.085	0.349 ± 0.17	0.013 ± 0.003	0.027 ± 0.006	0.114 ± 0.06	0.228 ± 0.121	0.0004 ± 0.0001	0.0008 ± 0.0002	0.0057 ± 0.004	0.011 ± 0.009	0.005 ± 0.0049	**0.011** ± 0.0098
	C	0.116 ± 0.057	0.233 ± 0.114	0.009 ± 0.002	0.018 ± 0.004	0.076 ± 0.04	0.152 ± 0.08	0.0003 ± 0.0001	0.0005 ± 0.0001	0.0038 ± 0.003	0.008 ± 0.006	0.004 ± 0.003	**0.007** ± 0.0065
P-DY	T	0.076 ± 0.023	0.152 ± 0.045	0.005 ± 0.001	0.010 ± 0.002	0.143 ± 0.078	0.285 ± 0.157	0.0002 ± 0.00003	0.0003 ± 0.0001	-		-	
	C	0.051 ± 0.015	0.102 ± 0.03	0.003 ± 0.0007	0.007 ± 0.001	0.095 ± 0.052	0.190 ± 0.104	0.0001 ± 0.00002	0.0002 ± 0.0001	-		-	
C-IC	T	0.093 ± 0.034	0.185 ± 0.067	0.022 ± 0.009	0.044 ± 0.018	0.126 ± 0.07	0.253 ± 0.14	0.0008 ± 0.0003	0.0015 ± 0.0005	-		0.0006 ± 0.0004	0.0011 ± 0.0009
	C	0.062 ± 0.022	0.123 ± 0.045	0.015 ± 0.006	0.029 ± 0.012	0.084 ± 0.047	0.169 ± 0.093	0.0005 ± 0.0002	0.001 ± 0.0003	-		0.0004 ± 0.0003	0.0008 ± 0.0006
V-IC	T	0.05 ± 0.014	0.10 ± 0.029	0.002 ± 0.0003	0.004 ± 0.0005	0.091 ± 0.055	0.182 ± 0.109	0.00004 ± 0.000003	0.00009 ± 0.00001	-		0.00015 ± 0.0001	0.0003 ± 0.00027
	C	0.033 ± 0.01	0.067 ± 0.019	0.0014 ± 0.0002	0.003 ± 0.0003	0.061 ± 0.036	0.121 ± 0.073	0.00003 ± 0.000002	0.00006 ± 0.000004	-		0.0001 ± 0.00009	0.0002 ± 0.00017
Tolerable daily intake		0.5^1^		0.07^1^		0.8^1^		0.0016^2^		0.3^3^		0.006^1^	

*T* toddlers, *C* children, ^1^EFSA (2024)^90^; ^2^EFSA (2012)^91^; ^3^EFSA (2014)^92^. The bold EDI indicated values above set TDI

**Table 4:** EDI (mg/kg_bw_/day) of probably essential and toxic trace elements through consumption of flavored dairy products (*n* = 180) in comparison to TDI/PTDI

Products	Consumer	Probably essential trace elements						Toxic trace elements							
		Mn		Ni		B		Pb		As		Al		Sb	
		1/D	2/D	1/D	2/D	1/D	2/D	1/D	2/D	1/D	2/D	1/D	2/D	1/D	2/D
C-UHT	T	0.024 ± 0.004	0.047 ± 0.007	-		0.021 ± 0.002	0.041 ± 0.004	0.0005 ± 0.0001	0.0011 ± 0.0002	-		0.027 ± 0.009	0.054 ± 0.019	-	
	C	0.016 ± 0.002	0.031 ± 0.005	-		0.014 ± 0.002	0.027 ± 0.003	0.0004 ± 0.0001	0.0007 ± 0.0001	-		0.018 ± 0.006	0.036 ± 0.012	-	
S-UHT	T	0.066 ± 0.026	0.132 ± 0.051	-		0.005 ± 0.005	0.011 ± 0.01	0.0015 ± 0.001	0.0029 ± 0.002	-		0.017 ± 0.007	0.034 ± 0.014	-	
	C	0.044 ± 0.17	0.088 ± 0.034	-		0.004 ± 0.003	0.007 ± 0.006	0.001 ± 0.0008	0.0019 ± 0.0015	-		0.011 ± 0.005	0.023 ± 0.009	-	
C-PM	T	0.039 ± 0.008	0.078 ± 0.016	-		0.080 ± 0.014	0.159 ± 0.027	0.0007 ± 0.0001	0.0014 ± 0.0002	0.00004 ± 0.00003	0.00008 ± 0.00006	0.025 ± 0.012	0.051 ± 0.024	-	
	C	0.026 ± 0.005	0.052 ± 0.011	-		0.053 ± 0.009	0.106 ± 0.018	0.0005 ± 0.00006	0.0009 ± 0.0001	0.00003 ± 0.00002	0.00005 ± 0.00004	0.017 ± 0.008	0.034 ± 0.016	-	
S-PM	T	0.035 ± 0.0017	0.069 ± 0.003	-		0.082 ± 0.004	0.165 ± 0.008	0.00009 ± 0.00007	0.00018 ± 0.0002	-		0.021 ± 0.01	0.042 ± 0.02	-	
	C	0.023 ± 0.001	0.046 ± 0.002	-		0.055 ± 0.003	0.110 ± 0.005	0.00006 ± 0.00005	0.00012 ± 0.0001	-		0.014 ± 0.007	0.028 ± 0.014	-	
C-MP	T	0.024 ± 0.0006	0.048 ± 0.001	-		0.011 ± 0.001	0.021 ± 0.003	0.0002 ± 0.00004	0.0004 ± 0.00008	0.00003 ± 0.00001	0.00005 ± 0.00003	0.014 ± 0.005	0.029 ± 0.010	-	
	C	0.016 ± 0.0004	0.032 ± 0.0008	-		0.007 ± 0.0009	0.014 ± 0.002	0.0002 ± 0.00002	0.0003 ± 0.00006	0.00002 ± 0.00001	0.00004 ± 0.00002	0.010 ± 0.003	0.019 ± 0.006	-	
S-MP	T	0.002 ± 0.0008	0.004 ± 0.0017	-		0.005 ± 0.001	0.010 ± 0.003	0.00002 ± 0.000003	0.00003 ± 0.00001	-		0.008 ± 0.002	0.016 ± 0.003	-	
	C	0.001 ± 0.0006	0.003 ± 0.001	-		0.003 ± 0.0009	0.006 ± 0.002	0.00001 ± 0.000002	0.00002 ± 0.000004	-		0.005 ± 0.001	0.010 ± 0.002	-	
S-Y	T	0.049 ± 0.027	0.098 ± 0.054	**0.024** ± 0.026	**0.048** ± 0.052	0.017 ± 0.004	0.035 ± 0.009	0.0014 ± 0.0005	0.0027 ± 0.001	**0.0006** ± 0.00047	**0.0011** ± 0.0009	0.044 ± 0.012	0.088 ± 0.023	-	
	C	0.033 ± 0.018	0.065 ± 0.036	**0.016** ± 0.017	**0.032** ± 0.035	0.012 ± 0.003	0.023 ± 0.006	0.0009 ± 0.0004	0.0018 ± 0.0007	**0.0004** ± 0.00031	**0.0008** ± 0.0006	0.030 ± 0.008	0.059 ± 0.015	-	
P-Y	T	0.039 ± 0.010	0.078 ± 0.021	-		0.024 ± 0.0007	0.049 ± 0.001	0.0009 ± 0.0004	0.0018 ± 0.0008	0.0003 ± 0.00004	**0.0006** ± 0.00008	0.030 ± 0.013	0.060 ± 0.026	-	
	C	0.026 ± 0.007	0.052 ± 0.014	-		0.016 ± 0.0005	0.032 ± 0.0009	0.0006 ± 0.0003	0.0012 ± 0.0005	0.0002 ± 0.00003	**0.0004** ± 0.00005	0.020 ± 0.009	0.040 ± 0.017	-	
S-DY	T	0.049 ± 0.031	0.097 ± 0.062	-		0.010 ± 0.006	0.02 ± 0.013	0.0023 ± 0.001	0**.0046** ± 0.002	0.00007 ± 0.00005	0.0002 ± 0.0001	0.082 ± 0.029	**0.164** ± 0.058	-	
	C	0.032 ± 0.021	0.065 ± 0.041	-		0.007 ± 0.004	0.013 ± 0.008	0.0015 ± 0.0007	0.0031 ± 0.0015	0.00005 ± 0.00004	0.0001 ± 0.00007	0.055 ± 0.019	0.110 ± 0.039	-	
P-DY	T	0.008 ± 0.001	0.017 ± 0.0026	-		0.031 ± 0.013	0.062 ± 0.026	0.0005 ± 0.00027	0.0011 ± 0.0005	-		0.089 ± 0.022	**0.177** ± 0.043	0.0005 ± 0.00056	0.0009 ± 0.0011
	C	0.006 ± 0.0009	0.011 ± 0.0017	-		0.021 ± 0.009	0.042 ± 0.017	0.0004 ± 0.00018	0.0007 ± 0.0004	-		0.059 ± 0.014	0.118 ± 0.029	0.00031 ± 0.00037	0.0006 ± 0.0007
C-IC	T	0.026 ± 0.011	0.053 ± 0.024	-		0.019 ± 0.005	0.037 ± 0.009	0.0008 ± 0.0006	0.0015 ± 0.0012	0.00007 ± 0.000067	0.0001 ± 0.0001	0.027 ± 0.012	0.054 ± 0.023	-	
	C	0.018 ± 0.008	0.035 ± 0.016	-		0.013 ± 0.003	0.025 ± 0.006	0.0005 ± 0.0004	0.001 ± 0.0008	0.00005 ± 0.000045	0.00009 ± 0.00009	0.018 ± 0.008	0.036 ± 0.016	-	
V-IC	T	0.004 ± 0.0007	0.008 ± 0.001	-		0.015 ± 0.008	0.030 ± 0.016	0.001 ± 0.0006	0.002 ± 0.001	-		0.014 ± 0.005	0.027 ± 0.009	0.000026 ± 0.000028	0.000052 ± 0.000056
	C	0.003 ± 0.0004	0.005 ± 0.0009	-		0.010 ± 0.005	0.020 ± 0.011	0.001 ± 0.0004	0.001 ± 0.0008	-		0.009 ± 0.003	0.018 ± 0.006	0.000017 ± 0.000019	0.000035 ± 0.000037
TDI		0.28^1^		0.013^2^		0.2^3^		0.0036^4^		0.0021^4^ 0.0003^5^		0.28^4^ 0.14^6^		0.006^7^	

*T* toddlers, *C* children, *TDI* tolerable daily intake,^1^EFSA (2023)^107^; ^2^EFSA (2020)^108^; ^3^EFSA (2024)^90^; ^4^PTDI provisional tolerable daily intake based on JECFA (2011)^97^; ^5^EFSA (2009)^95^; ^6^EFSA (2008)^94^; ^7^WHO (2011)^109^. The bold EDI indicated values above set PTDI

The uppermost aluminum daily intake values were estimated in strawberry- and peach-flavored drinking yogurt at 0.164 ± 0.058 and 0.177 ± 0.043 mg/kg_bw_/day, respectively, surpassing the TDI recommended by EFSA [94]. However, maximally calculated intakes for arsenic by toddlers in strawberry and peach yogurt samples were 0.0011 ± 0.0009 and 0.0006 ± 0.00008 mg/kg_bw_/day, respectively, exceeding EFSA [95] TDI (0.0003 mg/kg_bw_/day), as displayed in Table 4. This TDI of As set by EFSA [95] and by ATSDR [96] as MRL (minimum risk level) was used to evaluate the acceptability of arsenic EDI instead of PTDI announced by JECFA, as it has been withdrawn due to a lack of safety [97]. The tolerated weekly intake (0.025 mg/kg.bw.) of Pb stated was recently deemed unacceptable and considered hazardous, as this amount was found to possibly cause a lowering of 3 IQ degrees minimally in kids [98]. Various studies stated Pb EDI are higher than previously reported PTDI in milk consumed by infants and children by one serving daily, as noted by Capcarova et al. [99] and Boudebbouz et al. [25]. Filippini et al. [100] were notified of nearly comparable results for EDI of antimony, boron, and cobalt values of dairy products, with averages of 0.0004, 0.045, and 0.0011, respectively. However, milk powder samples showed a high metal content; it gave low EDI values due to the low amount needed for consumption. Probable water contamination was not involved in the investigation that required reconstitution, as further studies encouraged.

This result illustrates that routine consumption of products with unacceptable or nearly unacceptable contamination levels may suggest a potentially concerning toxic metals (Pb, As, Al, and Sb) exposure, especially for toddlers. The raw materials and compositions used in the preparation of flavored and fruit products, such as cacao powder and fruit purees, may be attributed to increasing element intake, as concluded by Chekri et al. [101] and de Paiva et al. [65]. The exposure of toddlers to vigorous neurogenic toxin aluminum metal for long periods with high consumption may expose them to prospective health dangers such as Alzheimer’s disease, osteomalacia, and severe nephritic disorders. In addition, recent studies suggested the contribution of Al in the development of breast cancer [66, 102, 103]. However, Pb and As are known to be responsible for probable intelligent inhibition and retardation [104, 105]. Nickel excessive intake may be linked to hormonal and enzymatic imbalance or cell injury [20]. While the cumulative oral exposure to Sb may be associated with genotoxicity, lowering sperm count, impairment of DNA synthesis, sugars and lipid metabolism, feeble embryotoxicity, and oral poisoning [106].

### The Non-cancer Health Risk Evaluation by Determination of THQ and TTHQ

**Fig. 2:**
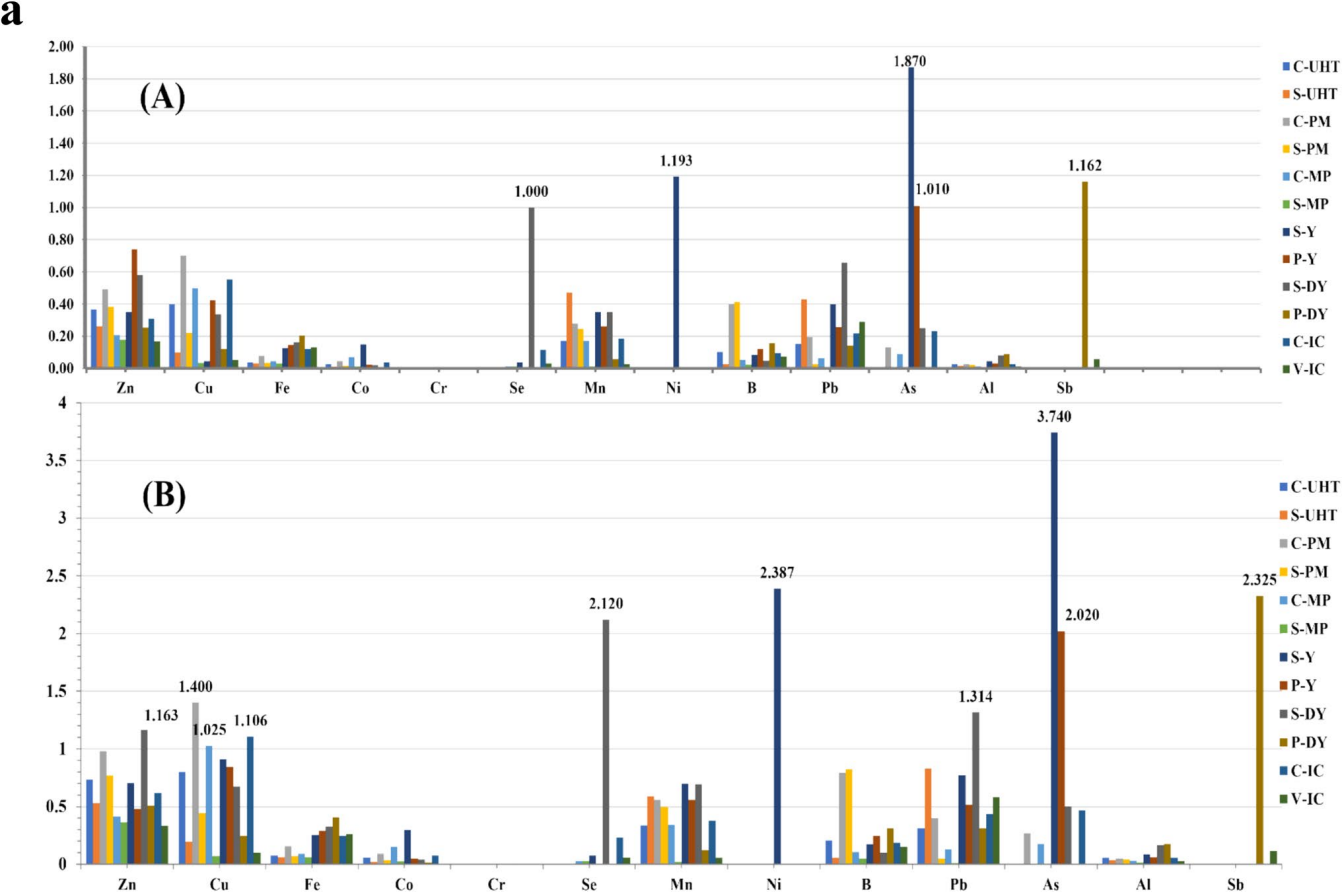

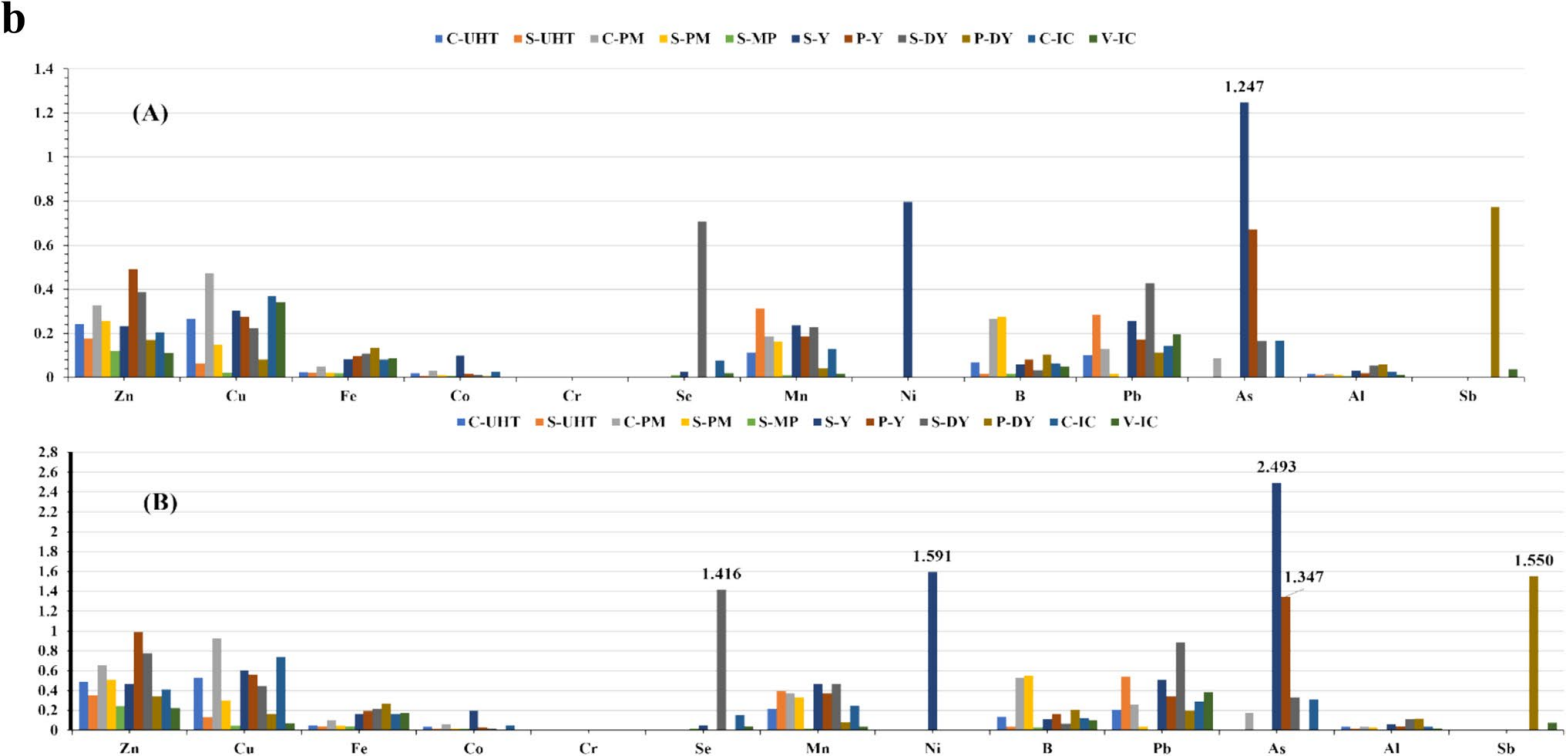
**a** THQ of each trace-metal caused by consuming of flavored dairy products for toddlers (A) Daily; (B) Twice daily. **b** THQ of each trace-metal caused by consuming of flavored dairy products for children (A) Daily; (B) Twice daily

Toddlers were found to be more susceptible to risk than children even in the lowest consumption, which was detected through arsenic in the consumption of the most preferable flavor—strawberry yogurt. By increasing the amount of consumption, the risk rises with a critical THQ value exceeding 1. The daily consumption of some products may cause non-cancer risks for toddlers such as arsenic and nickel in S-yogurt, arsenic in P-yogurt, antimony in P-drinking yogurt, and selenium in S-drinking yogurt, as well as arsenic for children via consuming S-yogurt. Consequently, on eating examined S-drinking yogurt twice a day, toddlers may suffer from Pb toxicity (THQ value of 1.314) as presented in (Fig. 2). The prolonged exposure to toxic As may give rise to cardiovascular disorders and developmental malformity with lowering intelligence quotient, but Pb may possess destructive effectiveness on varied systems such as urinary, digestive, immune, nervous, reproductive, skeletal, and cardiovascular [16, 104, 105]. However, non-carcinogenic diseased conditions associated with accumulated nickel exposure were heart and respiratory failure, asthma, allergic response, and giddiness [110]. Consequently, toddlers demonstrate to be the most vulnerable group due to lower body weight, increasing their risk of exposure [25, 111].

**Fig. 3:**
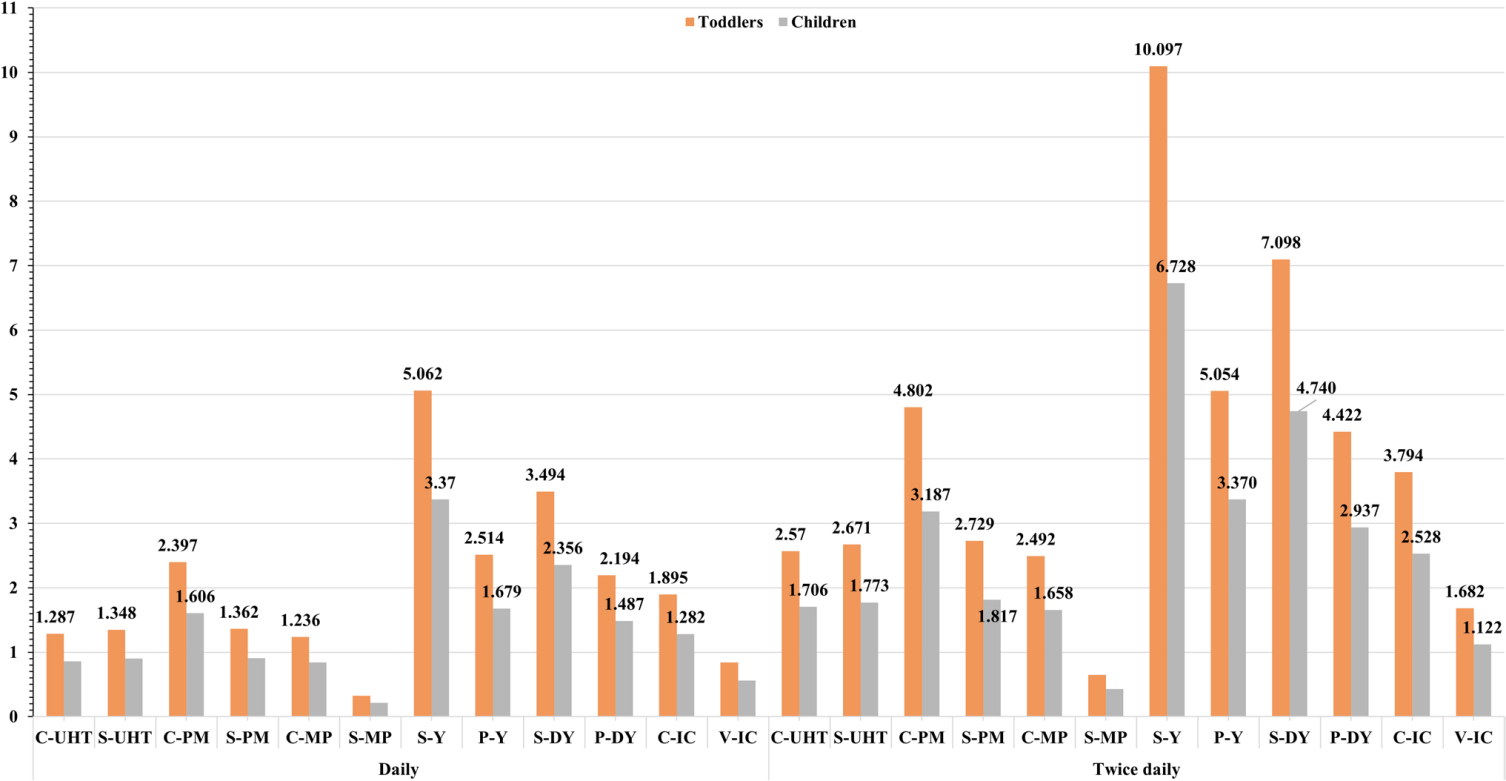
TTHQ of trace-metals caused by consuming of flavored dairy products for toddlers and children

Regarding the estimation of TTHQ, results were exhibited in Fig. 3; the considerable number of tested metals (15 metals) gives reliable value for TTHQ, which indicates the probability of health risk. The minimum consumption showed TTHQ values ranged from 3.37 for S-drinking yogurt in children to 5.062 for S-yogurt in toddlers. The results indicated that the main contributor to the elevated and riskiest TTHQ was fermented milk, as reported by Yu et al. [21]. Another recent study conducted by Boudebbouz et al. [25] recorded a TTHQ for milk consumption (9.30–27.89) greater than one, with the highest contribution by Pb (as a toxic metal), Ni (as a probably essential element), and Zn (as an essential element). Melo et al. [112] reported a THQ > 1 for Ni and As by consuming young child formula for kids (1–3 years) or fortified milk.

The other products that exhibited levels of TTHQ more than 1 of minimum consumption by toddlers are ranged as follows: C-pasteurized milk, C-ice cream, P-yogurt, and S-drinking yogurt in values of 2.397, 1.895, 2.514, and 3.494, respectively. The chronic exposure and accumulation of these toxic elements (Al, As, and Pb) in toddlers and children’s bodies may be the reason for serious thyroid and brain disorders, autism, or dying [113].

### The Cancer Healthiness Threat Assessment by Calculation of CR and TCR

**Fig. 4:**
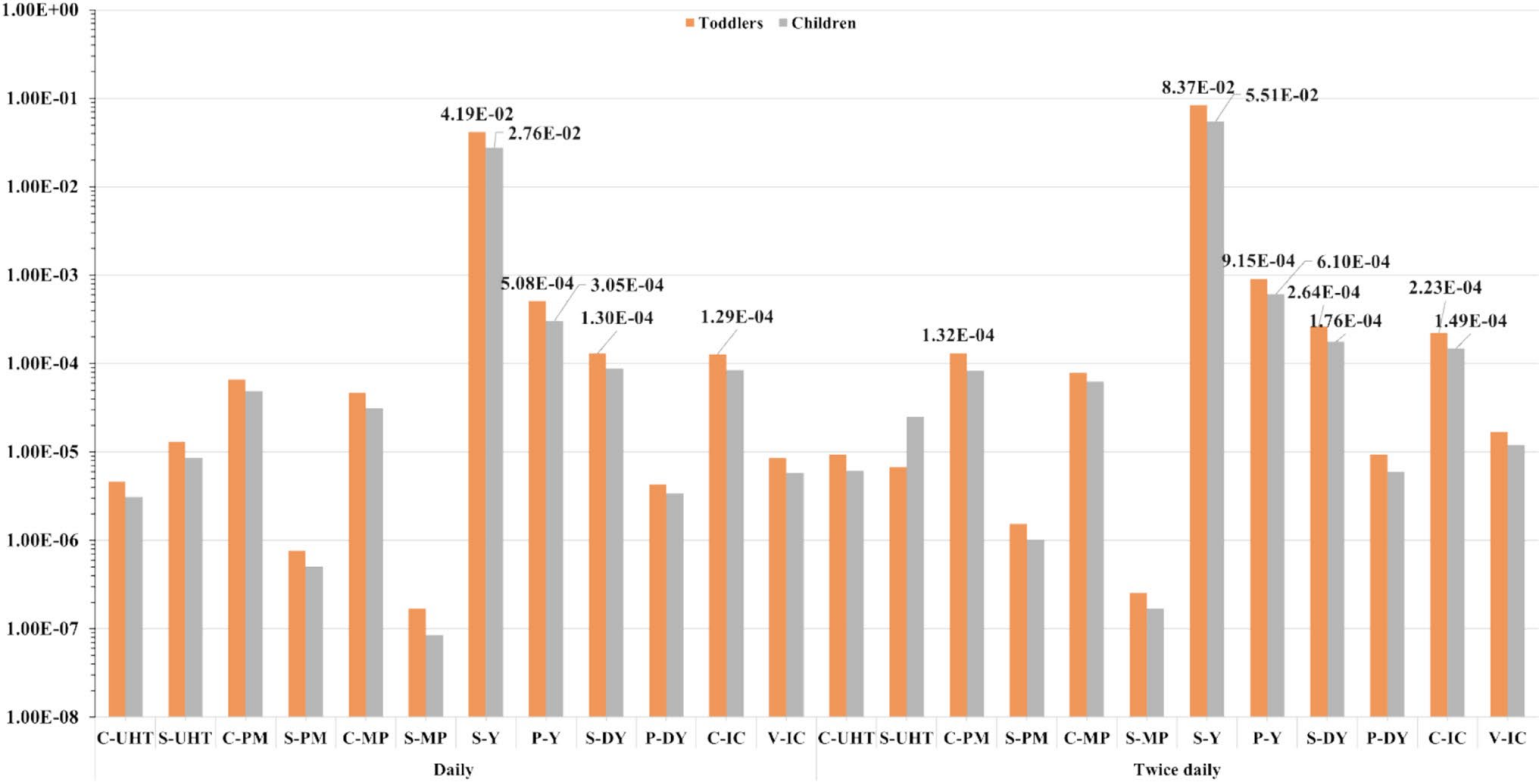
TCR of trace-metals caused by consuming flavored dairy products for toddlers and children

The cancer risk (CR) was estimated for Pb, As, and Ni for exhibition accumulative tolerability of cancer evolution as displayed in Table 5; the high CR value was detected in S-yogurt through Ni and As for both toddlers and children even on minimum consumption; therefore, it exhibited the maximum values of TCR (Fig. 4). TCR for toddlers and children in this product ranged from 4.19 × 10^−4^ to 8.37 × 10^−2^ and 2.76 × 10^−4^ to 5.5 × 10^−2^, respectively. The carcinogenicity of the three metals may be expressed in lung or bladder cancers, as well as arsenic as classified by ATSDR [96]; on the top of the carcinogen list, Group 1 poses a potential issue with chronic exposure causing several cancers in various organ forms such as kidney, liver, and skin [110, 114].

**Table 5:** CR of trace elements (Pb, As, and Ni) through consumption of flavored dairy products (*n* = 180)

Products	Consumer	Carcinogenic metals					
		Pb		As		Ni	
		Daily	Twice/day	Daily	Twice/day	Daily	Twice/day
C-UHT	T	4.60E-06	9.40E-06	-	-	-	-
	C	3.10E-06	6.10E-06	-	-	-	-
S-UHT	T	1.30E-05	2.50E-05	-	-	-	-
	C	8.50E-06	1.60E-05	-	-	-	-
C-PM	T	5.78E-06	1.19E-05	6.00E-05	**1.20E-04**	-	-
	C	3.83E-06	7.65E-06	4.50E-05	7.50E-05	-	-
S-PM	T	7.65E-07	1.53E-06	-	-	-	-
	C	5.10E-07	1.02E-06	-	-	-	-
C-MP	T	1.90E-06	3.40E-06	4.50E-05	7.50E-05	-	-
	C	1.28E-06	2.55E-06	3.00E-05	6.00E-05	-	-
S-MP	T	1.70E-07	2.55E-07	-	-	-	-
	C	8.50E-08	1.70E-07	-	-	-	-
S-Y	T	1.20E-05	2.30E-05	**8.40E-04**	**1.68E-03**	**4.10E-02**	**8.20E-02**
	C	8.00E-06	1.50E-05	**5.60E-04**	**1.13E-03**	**2.70E-02**	**5.40E-02**
P-Y	T	7.70E-06	1.53E-05	**5.00E-04**	**9.00E-04**	-	-
	C	5.10E-06	1.02E-05	**3.00E-04**	**6.00E-04**	-	-
S-DY	T	2.00E-05	3.90E-05	**1.10E-04**	**2.25E-04**	-	-
	C	1.30E-05	2.60E-05	7.50E-05	**1.50E-04**	-	-
P-DY	T	4.30E-06	9.40E-06	-	-	-	-
	C	3.40E-06	6.00E-06	-	-	-	-
C-IC	T	8.60E-06	1.28E-05	**1.20E-04**	**2.10E-04**	-	-
	C	4.30E-06	8.60E-06	8.00E-05	**1.40E-04**	-	-
V-IC	T	8.50E-06	1.70E-05	-	-	-	-
	C	5.80E-06	1.20E-05	-	-	-	-

*T* toddlers, *C* children, The bold CR indicated values above 10^−4^

The International Agency for Research on Cancer (IARC) has classified nickel (Ni) and lead (Pb) compounds as carcinogenic in Group 1 and probable carcinogens listed in Group 2B, respectively [115, 116]. The managing of trace element levels in dairy products is a necessity demanded for prospective toddlers and children’s health, especially for metals with cumulative effects on long-term exposure [23, 41, 117, 118]. A CR of arsenic was similarly recorded to exceed 10^−4^ with a possible risk for kids aged 1–3 by consuming fortified milk or young child formula [110]. The Pb results did not exhibit a carcinogenic tendency but were within secured limits (Table 5), nearly conformable to Muhib et al. [119] and Dhar et al. [89]. Though arsenic and nickel results may demonstrate potential cancer risk, CR is a beneficial tool for recognizing the health issues of their exposures and how to improve decision creation for achieving a minimal CR index.

## Conclusion

Flavored dairy products perform a remarkable challenge role in daily consumption for toddlers and children to achieve their nutrient neediness. In this current study, recent valuable data was obtained concerning 15 trace elements for the first record in flavored various dairy products (UHT, pasteurized, powdered, fermented milk, and ice cream). The Zn and Fe, followed by Mn and Cu, were the maximal essential elements measured in dairy products, with Pb content exceeding the regulatory limit in UHT, C-PM, C-MP, fermented milk, and ice cream. However, values for arsenic did not surpass the WHO limit; it exhibited an EDI for daily and twice daily consumption in flavored yogurt with the highest THQ in both toddlers and children. Therefore, we cannot depend on one parameter for the risk assessment of products’ metal content, but a full evaluation was recommended, such as comparing concentration with available international regulations, which required reevaluation and renovation, estimation of THQ and TTHQ, and computing of CR and TCR. Although to eliminate or diminish the toxic element content in target products, we should take into consideration the safety concept of farm-to-fork with exhaustive monitoring and evolving in the industrial steps or techniques as well. In addition, daily intake, mainly for toddlers of tasty milk products, should be observed and monitored by increasing their mothers’ awareness.

Furthermore, organized monitoring and data fulfillment are recommended and proposed substantially for metal contents and influence assessment in future research studies. Taking into consideration the other possible sources of metal contamination, like water used for milk powder reconstitution and other food sources rather than milk products, as well as studies including the evaluation of raw material used in processing involved in the manufacturing plant to more specifically identify the highest origin of contamination, is recommended. Also, improving cattle feed quality is recommended with remediation of soil and water metal contamination, which is regarded as a preventive approach.

## Supplementary Information

Below is the link to the electronic supplementary material.

Supplementary file1 (DOCX 26 KB)

## Data Availability

No datasets were generated or analysed during the current study.
