# Supplementary material for: Dietary Exposure and Health Risk Assessment of Selected Toxic and Essential Metals in Various Flavored Dairy Products
Source: Biol Trace Elem Res. 2025 Jun 4;204(1):218–38. doi: 10.1007/s12011-025-04676-0 (PMC12847127; doi:10.1007/s12011-025-04676-0)
Supplement: Supplementary file 1 — Supplementary file1 (DOCX 26 KB) [file 12011_2025_4676_MOESM1_ESM.docx]

**Table S1** Operating condition of ICP-MS/MS (Agilent 8800) and type gases used

| **RF power (W)** | 1550 |
| --- | --- |
| **Nebulizer (carrier) gas flow (L/min)** | 0.98 |
| **auxiliary gas flow (L/min)** | 0.8 |
| **Plasma gas flow (L/min)** | 15 |
| **Cell gas flow (mL/min)** | He 5.1  O_2_ 0.45 |
| **Dwell time (ms)** | 50 |
| **Sample uptake rate (s)** | 45 |
| **Replicates per Sample** | 3 |
| **Type of gases** | |
| Argon gas | For plasma ignition and maintenance of stable plasma |
| Helium (He) | Collision gas as used for Kinetic Energy Discrimination (KED) mode to remove polyatomic interferences by collisional energy loss which is especially effective in eliminating ArCl⁺ interference from ^75^As⁺. |
| Hydrogen (H₂) | Reaction gas for Neutralizing interferences by charge transfer or protonation. |
| Oxygen (O₂) | Reaction gas forms oxide product ions of target analytes or interferences. |

**Figure S1** Flow diagram of health risk assessment of toxic and essential metals determination in various flavored dairy products
